# Supplementary material for: MetaRibo-Seq measures translation in microbiomes
Source: Nat Commun. 2020 Jun 29;11:3268. doi: 10.1038/s41467-020-17081-z (PMC7324362; doi:10.1038/s41467-020-17081-z)
Supplement: Supplementary file 10 — Supplementary Data 7 [file 41467_2020_17081_MOESM10_ESM.zip › File2/Confidence_High_Taxonomy/386435_out.krona.html]

Javascript must be enabled to view this page.

members
magnitude
magnitudeUnassigned
count
unassigned
taxon
rank

386435\_out

72

superkingdom
72
2

72
phylum
976

class
72
200643

171549
72
order

72
family
815

72
genus
816


SRS143417\_contig\_number\_28075
species
1
46506

818
1
species

SRS075878\_contig\_number\_347

2292951

SRS143417\_contig\_number\_3704
species
1

817
species

SRS011084\_contig\_number\_1620SRS014613\_contig\_number\_1748SRS014736\_contig\_number\_309SRS015264\_contig\_number\_12459SRS015854\_contig\_number\_17603SRS016095\_contig\_number\_11980SRS016132\_contig\_number\_642SRS016267\_contig\_number\_1762SRS017521\_contig\_number\_28381SRS017622\_contig\_number\_212SRS018541\_contig\_number\_10415SRS018656\_contig\_number\_12095SRS018888\_contig\_number\_4945SRS018984\_contig\_number\_1659SRS019397\_contig\_number\_590SRS023829\_contig\_number\_1501SRS024075\_contig\_number\_9975SRS024388\_contig\_number\_2497SRS024549\_contig\_number\_656SRS043411\_contig\_number\_15598SRS046502\_contig\_number\_12906SRS046712\_contig\_number\_667SRS048981\_contig\_number\_175SRS049402\_contig\_number\_8694SRS050299\_contig\_number\_3991SRS051610\_contig\_number\_9365SRS053214\_contig\_number\_6608SRS055966\_contig\_number\_1223SRS058723\_contig\_number\_2620SRS063040\_contig\_number\_25575SRS063127\_contig\_number\_2570SRS063190\_contig\_number\_11052SRS064276\_contig\_number\_5774SRS075021\_contig\_number\_179SRS075773\_contig\_number\_23524SRS076804\_contig\_number\_13576SRS077194\_contig\_number\_13266SRS077589\_contig\_number\_929SRS077730\_contig\_number\_15327SRS097920\_contig\_number\_19545SRS098644\_contig\_number\_11966SRS098655\_contig\_number\_299SRS098881\_contig\_number\_15244SRS104036\_contig\_number\_7946SRS104311\_contig\_number\_19447SRS104485\_contig\_number\_1147SRS1054928\_contig\_number\_3096SRS1055043\_contig\_number\_9030SRS1055069\_contig\_number\_11728SRS140492\_contig\_number\_22200SRS140513\_contig\_number\_11253SRS142599\_contig\_number\_17807SRS143417\_contig\_number\_301SRS143598\_contig\_number\_5436SRS144135\_contig\_number\_30282SRS144362\_contig\_number\_20359SRS144506\_contig\_number\_21475SRS146812\_contig\_number\_53853SRS147022\_contig\_number\_2426SRS147272\_contig\_number\_398SRS147377\_contig\_number\_3759SRS147557\_contig\_number\_31189SRS147653\_contig\_number\_7304SRS148159\_contig\_number\_56570SRS893279\_contig\_number\_8136SRS893342\_contig\_number\_5885SRS971275\_contig\_number\_17111
67

329854
species

SRS104636\_contig\_number\_15531SRS147139\_contig\_number\_30055
2
